# Reforming Medical Education in Pakistan through strengthening Departments of Medical Education

**DOI:** 10.12669/pjms.346.15942

**Published:** 2018

**Authors:** Muhammad Zahid Latif, Gohar Wajid

**Affiliations:** 1 *Dr. Muhammad Zahid Latif, MBBS, MPH, MME, PhD (Scholar). Department of Community Medicine & Medical Education, Azra Naheed Medical College, The Superior University, Lahore, Pakistan*; 2 *Dr. Gohar Wajid MBBS, MSc, MPH, PhD. Advisor Medical Education, The University of Lahore, Lahore, Pakistan*

**Keywords:** Department of Medical Education, Medical Education, Medical Colleges, Faculty Development

## Abstract

**Objective:**

To review the current status of departments of medical education in all public and private medical colleges located in the city of Lahore, Pakistan.

**Methods:**

This was a quantitative, cross sectional descriptive study; conducted from March to October 2015 in Pakistan Medical & Dental Council (PM&DC) recognized medical colleges located in Lahore, Pakistan. Respondents were the heads of departments of medical education or any other well-informed faculty member. A questionnaire was prepared to obtain information about the current status of the departments of medical education (DMEs). The investigator personally visited all medical colleges for data collection. Both verbal and written consents were obtained and the questionnaire was administered to the resource persons. The data was organized and entered in SPSS for descriptive analysis.

**Results:**

Out of the 18 medical colleges in Lahore, six (33.3%) belonged to public sector and 12 (66.7%) were from private sector. All medical colleges reported to have a functional DME. However, eight had established DMEs during the past five years. Only one (5.6%) head of DME was working on full-time basis. Eleven (61.1%) heads of DMEs did not have any formal qualification in medical education. Eight (44.4%) colleges claimed to have adequate human resources for DME. Thirteen (72.2%) colleges mentioned that adequate financial resources were available for running DMEs. It is encouraging to see that DMEs in private sector medical colleges are playing increasingly significant role in managing educational activities. Similarly, the senior management of private sector seems to be relatively more eager to promote educational activities.

**Conclusion:**

There is an increasing recognition towards establishing DMEs in the medical colleges, but their infrastructure, proper functioning and availability of human and financial resources are serious impediments requiring immediate attention.

## INTRODUCTION

Global educational advancements have significantly changed the face of medical profession, making teaching and learning more relevant to societal needs.^1^ Modern medical education is expected to meet the requirements of local and universal communities.^2^,^3^ While health and education may be highly contextualized, the latest trends in medical education in developing countries are derived mainly from the West and may not always take account of the local context.^2^,^4^ The diversity of cultures, values, epidemiological and demographic characteristics stipulate for localization of these concepts. The past few decades have witnessed numerous trends in transforming medical education including the development of educational frameworks, competency-based education and increased demands for compassion and care from the healthcare providers.^5^ Globalization has intensely jolted the process of medical education leading to a more complex procedure for the development of physicians.^6^ The demands for the professionalization and need for strengthening educational regulations has intensified. The launch of global standards by the World Federation for Medical Education (WFME) was an initial step towards standardization.^7^ Similarly, the Educational Commission for Foreign Medical Graduates declared that by 2023, only those candidates will be allowed having graduation from a program accredited by WFME or other global criteria for an accrediting body.^7^ The emerging educational scenario has raised the demand for professionalization of medical education and the need for strengthening the educational regulations.^2^

Today, there is an increasing realization that the complex medical education system be driven by professional educationists with well-established educational infrastructure in medical colleges. There is an escalating trend for the establishment of departments of medical education (DME) (also called departments of health professions education and educational development units). These departments form essential component of infrastructure to provide effective and high quality educational services in health professions education institutes. The scope of these departments has been well recognized and few important functions include faculty development, educational research, curriculum development and monitoring, student assessment and evaluation of teaching and learning activities.^8^

Medical education has advanced rapidly in Pakistan over the past few decades. Public sector dominated the educational scenario till 1990, with around 20 medical and dental colleges while only two were available in private sector. The balance however started tilting towards private sector from early 1990s. Today, there are 88 colleges available in private while only 48 in public sector.^9^ Exponential increase in the number of medical and dental colleges, especially in the private sector has raised serious concerns about the quality of medical education and the accreditation system developed to recognize these colleges. Out of 136 medical and dental colleges operating in the country now, 112 have been opened in the past 25 years.^9^ Although there are few improvements, still most of these colleges (especially in the public sector) are still following teacher centered, traditional subject based curricula, being managed by teachers with little formal training in teaching and learning. The establishment of medical education departments is considered a significant milestone towards improving the quality of medical education in these colleges.

Until recently, in Pakistan, there was no concept of managing education in medical and dental colleges through well-organized departments of medical education. The pioneering institutes involved in the establishment of departments of medical education include King Edward Medical College (1973), College of Physicians & Surgeons Pakistan (1979), University of Health Sciences (2004), Khyber Medical University (2012) and Dow University of Health Sciences (2010).^10^-^12^ The private sector universities including Aga Khan University (1987), Baqai Medical University (1993), Ziaudin University (1996), Riphah International University (2005) and The University of Lahore (2012), also took significant initiatives for the development of medical education.

Establishing departments of medical education gained serious attention when PM&DC issued directives to establish these departments in all medical colleges in 2008.^8^. However, the PM&DC (as the national regulatory body) did not provide the much needed regulations, policy guidelines and technical support on the establishment, role and responsibilities of these departments. In the absence of such support, the role of these departments remains ambiguous. Most medical colleges are struggling to develop DMEs amidst chaos and confusion on how should they be established and what should be their role and functions. There is a shortage of qualified and trained medical educationists to take up the challenges of establishing these departments and operationalizing them to their optimum performance. This research was conducted to review the current status of DMEs in all public and private medical colleges located in the city of Lahore, Pakistan.

## METHODS

A quantitative, cross sectional, descriptive study was designed to review the status of DMEs in all medical colleges located in Lahore. Ethics approval was obtained from the Institutional Ethical Review Board (IERB) at The University of Lahore. All medical colleges recognized by PM&DC (n=18), located in the city of Lahore were included in the study. Respondents were the heads of department of medical education or any other well-informed faculty member in the medical college. A questionnaire was prepared to obtain information about the current status of DMEs in terms of their structure and functioning. The first version of the questionnaire was developed by the authors, after reviewing relevant literature that could help in questionnaire development. This questionnaire was discussed with subject experts for the adequacy and clarity of content, ease of availability of information and the construction and format of the questionnaire. Necessary changes were made in view of the expert opinion. Final version of the questionnaire comprised of 20 questions, divided into four sections. The first section consisted of the basic information about the respondent, establishment of the college and the DME. The second section assessed the availability of human resources, financial resources and infrastructure for the DME. The third section appraised the role of DME in the college and the commitment of senior management to improve medical education. The last section consisted of open ended questions about the barriers to improving the quality of work at DME and suggestions to improve it. It was also pilot tested in two institutes outside the sampling area and modifications were made accordingly.

The investigator visited all medical colleges for data collection during March to October 2015. Respondents were assured about confidentiality. Both verbal and written consents were obtained and the questionnaire was administered to the resource persons. Average time required to fill the questionnaire was 25 minutes. The investigator also prepared a questionnaire-based check list for the verification of the provided data. This check list was filled by the investigator (MZL) after visiting each DME included in the study. The collected data was organized and entered in version 16 of Statistical Package for Social Sciences (SPSS) for analysis.

## RESULTS

Among all PM&DC recognized medical colleges located in Lahore, six (33.3%) were from the public sector and 12 (66.7%) belonged to private sector. Data was collected from all DMEs, giving a response rate of 100%. All medical colleges reported to have a functional DME, however the types of functions performed by them and their quality varied. Eight out of 18 (44.4%) medical colleges had established DMEs during the past five years only. Only in one (5.6%) head of DME was working on full^-^time basis. Seventeen medical colleges had part time heads of DME, 50% were full time clinicians and 44.4% belonged to basic sciences. Eleven (61.1%) heads of DMEs, out of 18 did not have any formal qualification in medical education, indicating the lack of qualified staff to run DMEs. Eleven (61.1%) DMEs did not have any information available on the college website.

Regarding the availability of infrastructure for DMEs, 16 (88.8%) medical colleges had at least one room dedicated for this purpose. Only eight (44.4%) colleges reported to have adequate human resources available for DME. There was no significant difference between the private and public sector medical colleges about the availability of human resource. Thirteen (72.2%) medical colleges reported that adequate financial resources were allocated for DME functions. All private sector medical colleges reported to have adequate finances available for DME while only two public sector medical colleges mentioned having adequate financial resources. The term ‘adequate’ however remains ambiguous due to lack of PM&DC guidelines on human and financial resources.

**Fig.1:**
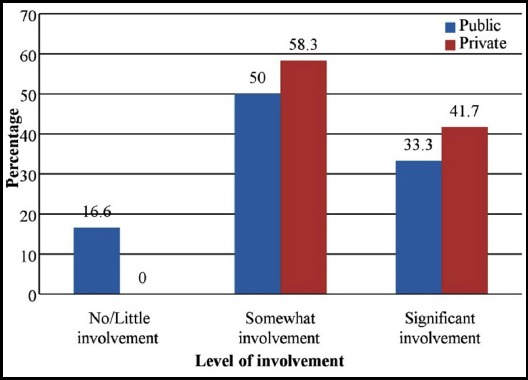
Involvement of department of medical education in planning and organizing educational activities

**Fig.2:**
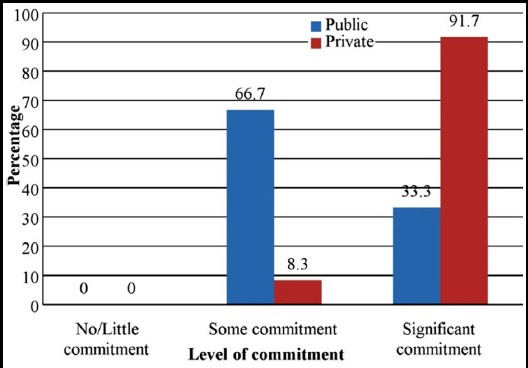
Commitment of senior management to promote medical education in the colleges

Respondents were asked about the involvement of DME in educational activities of the college. As evident from Fig.1, DMEs in private sector medical colleges are playing increasingly significant role in managing educational activities. When asked about the role of senior management in promoting educational activities, once again, private sector showed more serious commitment than public sector colleges (Fig. 2).

Respondents were asked about the most common functions performed by the DMEs. Table-I presents the list of most frequently reported functions. Faculty development (77.7%), curriculum development (77.7%), assessment of students (72.2%) and educational research (66.6%) included the top four functions respectively. Participants were asked about the barriers to improving DMEs in the colleges. The list of most frequently mentioned barriers is shown in Table-II. As perceived by the respondents, the three major barriers included lack of infrastructure and resources (72.2%), lack of qualified and trained human resources (66.6%) and resistance from faculty and top management (61%). Participants were asked to recommend major steps for the improvement of medical education in their colleges. Sixteen participants suggested the need for serious commitment from top leadership, better coordination with PM&DC and recruitment of full time, qualified and trained staff as major steps towards developing DMEs in the colleges.

**Table-I:** Distribution of functions of departments of medical education in the medical colleges of Lahore.

Functions	Frequency	Percentage
Faculty development	14	77.7
Curriculum development	14	77.7
Assessment	13	72.2
Research	12	66.6
Teaching & learning	5	27.7
Timetable setting	2	11.1
Mentoring/ student support	2	11.1

**Table-II:** Major barriers to improving medical education in medical colleges of Lahore.

Barrier	Frequency	Percentage
Lack of infrastructure and resources	13	72.2
Lack of qualified and trained HR	12	66.6
Resistance from faculty and top management	11	61
Lack of authority and coordination with PM&DC	7	38.9

## DISCUSSION

Departments of medical education are considered as a mandatory requirement for the accreditation of a medical college with PM&DC.^8^,^13^ The results of the current study reveal that all medical colleges in Lahore had DMEs which is in line with the requirement of PM&DC. However, 44.4% of the DMEs were established during the past five years, reflecting an increased recognition about their importance. Globally, establishment of these departments in medical institutes is related with the increased public expectations about health care, demands for social accountability, educational developments and the need to train more doctors.^14^ Although it was reported by all colleges that they had a functioning DME, but the findings about the job status of the head of DME revealed a different scenario. Only one (5.6%) head of DME was working on full-time basis and eleven (61.1%) did not have any formal qualification in medical education. The results are contrary to the requirement of a functional DME mentioned by Davis et al. in the guide for establishment of a medical education department.^14^ The head of DME is expected to exhibit leadership qualities to have clear vision about promoting medical education, envisaging educational challenges and managing the change process amidst, at times, stern faculty resistance. Leading and managing a DME is a full-time job and the head is expected to motivate and stimulate the lively exchange of ideas among faculty and should have the vision to bridge the current and future state of education in the institute.^15^,^16^ Different attributes of a director of educational program have been included in literature including, visionary, flexible, open-minded, trustworthy and value-driven.^17^ The head of DME working on part time basis may have difficulty in meeting the versatile requirements of the department. One of the issues in Pakistan is lack of recognition that head of DME can be a PhD in medical education and may not necessarily be a medical doctor.

Our study shows that only one DME was being led by a full time and qualified medical educationist. This shows the suppressive state of DMEs in the existing scenario and is contrary to the requirements of academic staff for a DME having a variety of academic backgrounds, including PhDs in HPE or other educational qualifications.^18^ The situation is likely to improve as medical education leadership in the country gains maturity and the profession exhibits its boundaries more assertively. Clinicians and basic sciences teachers working in DMEs on part time basis should be replaced with full time qualified educationists.

In our study, only eight (44.4%) medical colleges reported to have adequate human resources for DME. Ironically, the staff working in DMEs was neither qualified nor trained in medical education, except one college. Adhocism among medical education staff clearly prevails in Pakistani institutions. The results are different from the recommendations by Davis et al. for the staffing of DMEs which describe that a multi professional team from different professional backgrounds including medical and educational expertise is a basic requirement for running a well-functioning DME.^14^ An ideal skill mix of human resource for a DME includes health professionals, organizers, thinkers, innovators and motivators.^14^ DMEs are likely to start performing better once PM&DC issues policy for staff requirements and strictly enforces regulations to employ full time and qualified educationists.

Good teaching whether conducted in the class room, community or hospital requires adequate financial resources. A study by Knapp about financing of medical education describes that inadequate financing is one of the major problems of the medical education.^19^ Innovative approaches to teaching, development of skills, high quality assessment and development of professionalism require resources in terms of time and finances.^20^ Similarly, famous medical education historian Ludmerer concludes that the mission of educational improvement in medical schools is very expensive.^21^ In our study, 13 (72.2%) medical colleges reported having adequate financial resources for the DME. However, information about the allocation of actual funds was not available. The distribution of financial allocation may vary between the private and public-sector colleges. These departments are likely to get more funds once they start showing a positive impact on improving the quality of medical education in the colleges. A relevant study about the role of medical education and expectations of the faculty in a medical college of Lahore recommends to convince the management for the provision of funding for the initial years until the department becomes self-supporting.^11^

DMEs are considered as “a must department” to play crucial role in the planning and implementation of educational activities in the colleges.^22^ A DME should have an all-embracing function including teaching, research, and service provision and nurturing the carriers of the academic staff. However, the balance of these activities may vary in individual departments. Participants were asked about the involvement of DMEs in educational planning and implementation as ‘no/little involvement, somewhat involvement and significant involvement’ (Fig.1). Only seven DMEs (two from public and five from private sectors) had ‘significant’ involvement in planning and organizing medical education activities. Ten others showed ‘somewhat’ involvement. Results indicate that DMEs are paving their ways to influencing medical education in the colleges amidst lack of leadership and technical staff, scanty financial resources and unclear role and responsibilities.

Albanese et al. state that the senior management including deans, once committed, can promote the activities of DMEs in a systematic and coordinated manner.^23^ In our study, respondents were asked to respond to the level of commitment of the senior management in promoting medical education (Fig.2). Eleven out of 12 colleges in the private sector reported ‘significant’ commitment, while only two out of six colleges in the public sector mentioned the same level of commitment. Apparently, private sector is taking more interest in promoting medical education in the country, perhaps due to anticipated competition in improving the quality of medical education. The role of the PM&DC in leading and regulating medical education and using DMEs effectively to assure the quality of medical education becomes even more critical.

Following global trends, there is rapid change in health professionals’ education scenario in Pakistan. The number of educational institutes including medical universities is on the rise, posing new threats to the quality of medical education.^24^ Modern educational concepts such as curricular integration, better assessment methods, social accountability of educational institutes, inter-professional education/collaboration, educational informatics and introduction of more robust accreditation standards are rapidly taking their roots. The need for well-staffed and actively functioning DMEs is increasingly being realized not only at the undergraduate and postgraduate levels, but also at the level of regulatory bodies and policy making institutes such as PM&DC and Higher Education Commission. Our study shows that most DMEs in Lahore have been opened in recent past. The staff running these departments mostly does not have a formal qualification in medical education. Functions of DME are also unclear. This leads to a situation where most DMEs find it difficult to identify their role and create space for their effective functioning.

Given a chance, DMEs can perform a range of functions and provide technical support on issues such as educational leadership, governance and management of educational processes at the PM&DC, HEC, medical universities and undergraduate medical college levels. As the central regulatory body in the country, PM&DC has key role to play in effectively engaging medical institutes and providing them technical support to establishing DMEs and improving the quality of medical education through their effective functioning. In the absence of such support the purpose and role of these departments remains elusive.

## CONCLUSION

There is increasing recognition towards establishing departments of medical education in the medical and dental colleges, but their infrastructure, proper functioning and availability of financial resources are serious issues. Majority of colleges do not have a full time, qualified director/ head and adequate human and financial resources. Well established and properly functioning DMEs have the ability to play effective role in improving the quality of medical education. PM&DC, HEC and medical universities should provide leadership and guidance on policy, governance and regulatory issues and strengthen technical capacity of these departments at medical and dental colleges in Pakistan.

### Authors’ Contribution

**MZL:** Conceived, designed, collected data, analysis and write up.

**GW:** Research design, edited the manuscript& reviewed and finalized.
